# Supplementary material for: Tegaserod Maleate Suppresses the Growth of Gastric Cancer In Vivo and In Vitro by Targeting MEK1/2
Source: Cancers (Basel). 2022 Jul 23;14(15):3592. doi: 10.3390/cancers14153592 (PMC9332868; doi:10.3390/cancers14153592)
Supplement: Supplementary file 1 [file cancers-14-03592-s001.zip › cancers-1794434-supplementary.pdf]

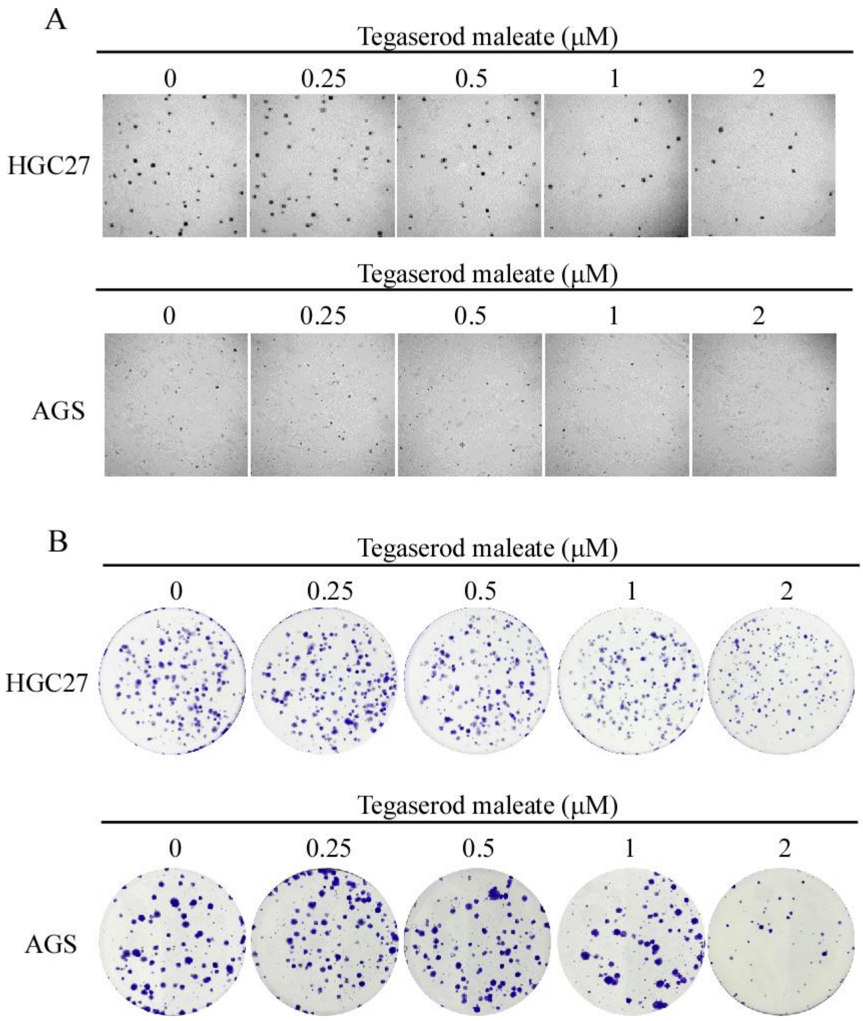

Figure S1. (A)The representative pictures of anchorage-independent cell clones. (B)The representative pictures of anchorage-dependent cell clones.

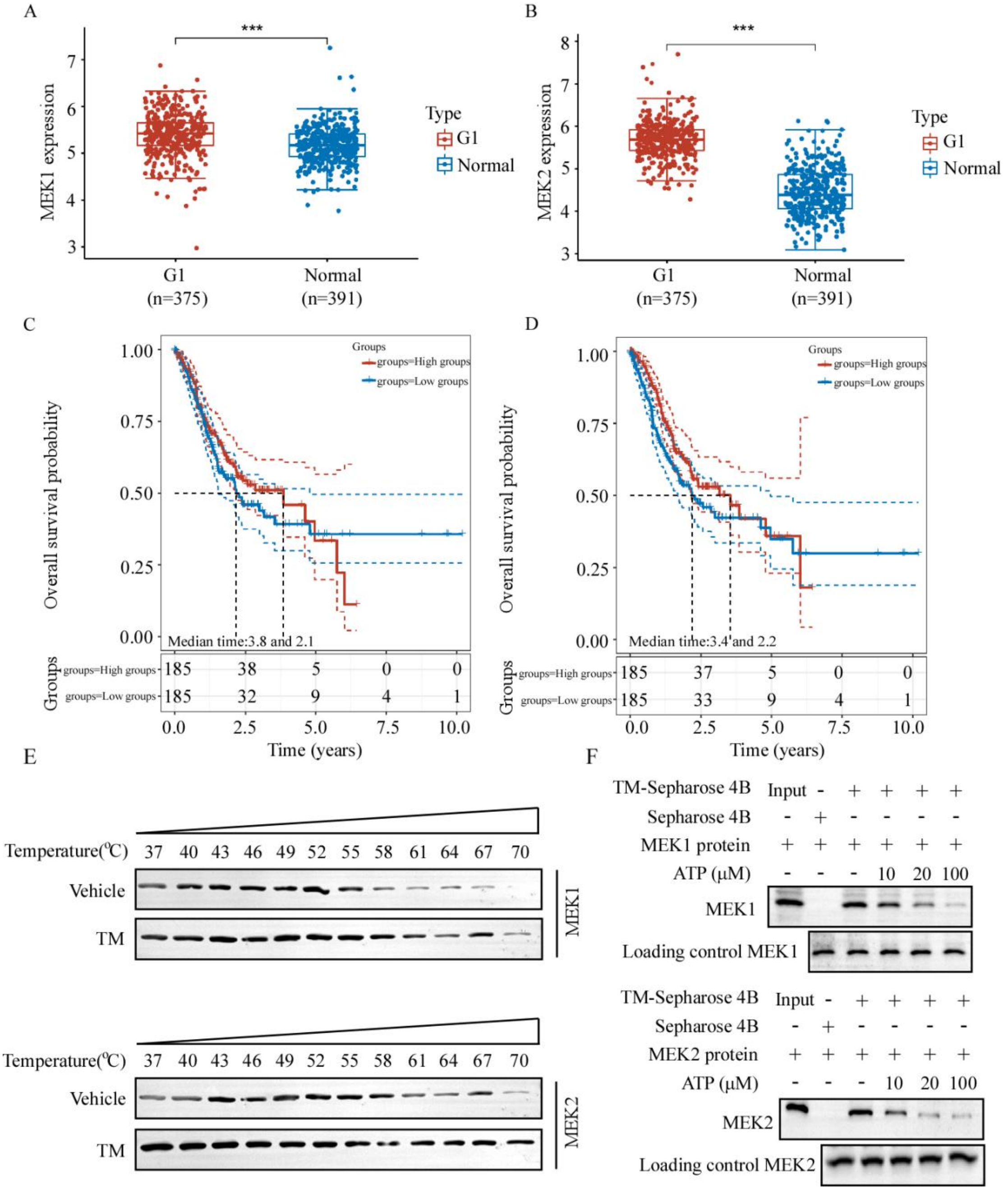

Figure S2. (A) The protein levels of MEK1 in GC from the TCGA database. (B)The protein levels of MEK2 in GC from the TCGA database. (C) Kaplan–Meier curves of overall survival in patients with MEK1 high expression. (D) Kaplan–Meier curves of overall survival in patients with MEK2 high expression. (E) The representative pictures of Western blotting for CETSA. (F)The specificity of the binding of tegaserod meleate to active MEK1 and MEK2 in the presence of ATP was evaluated. The asterisks (***) indicate a significant ($p < 0.001$).

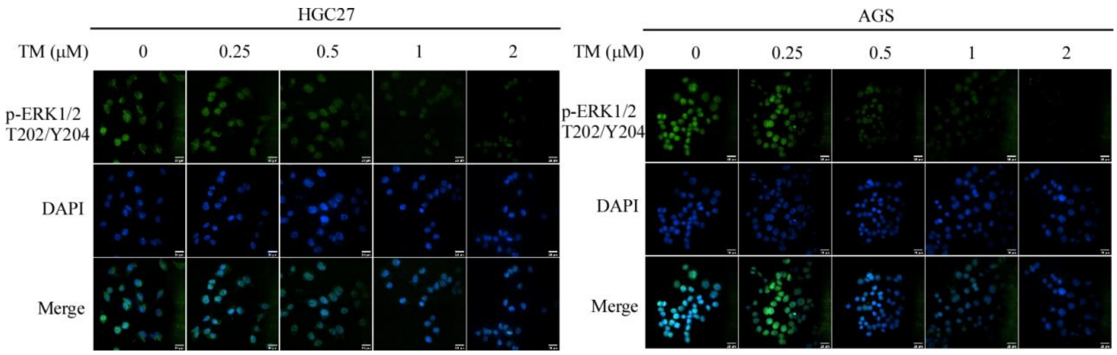

Figure S3. The representative pictures of immunofluorescence.

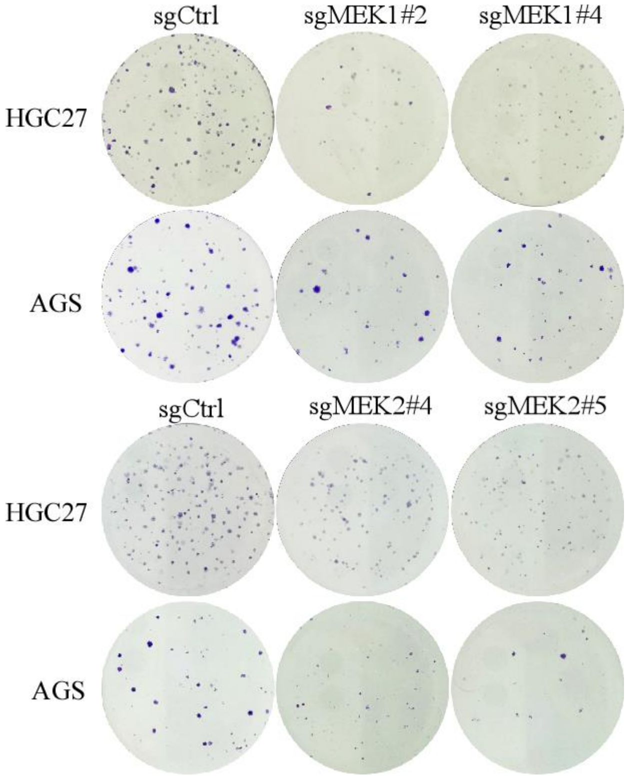

Figure S4. The representative pictures of clones after MEK1 or MEK2 knockout.

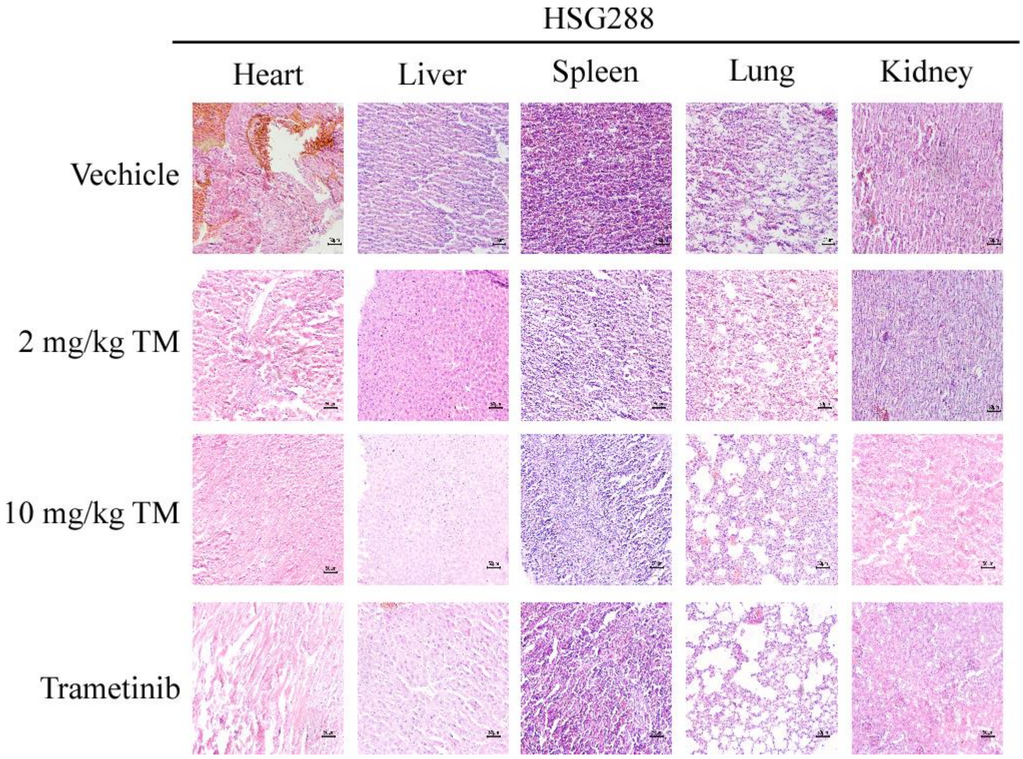

Figure S5. The HE staining on the heart, liver, spleen, lung and kidney of HSG288 PDX mouse model.

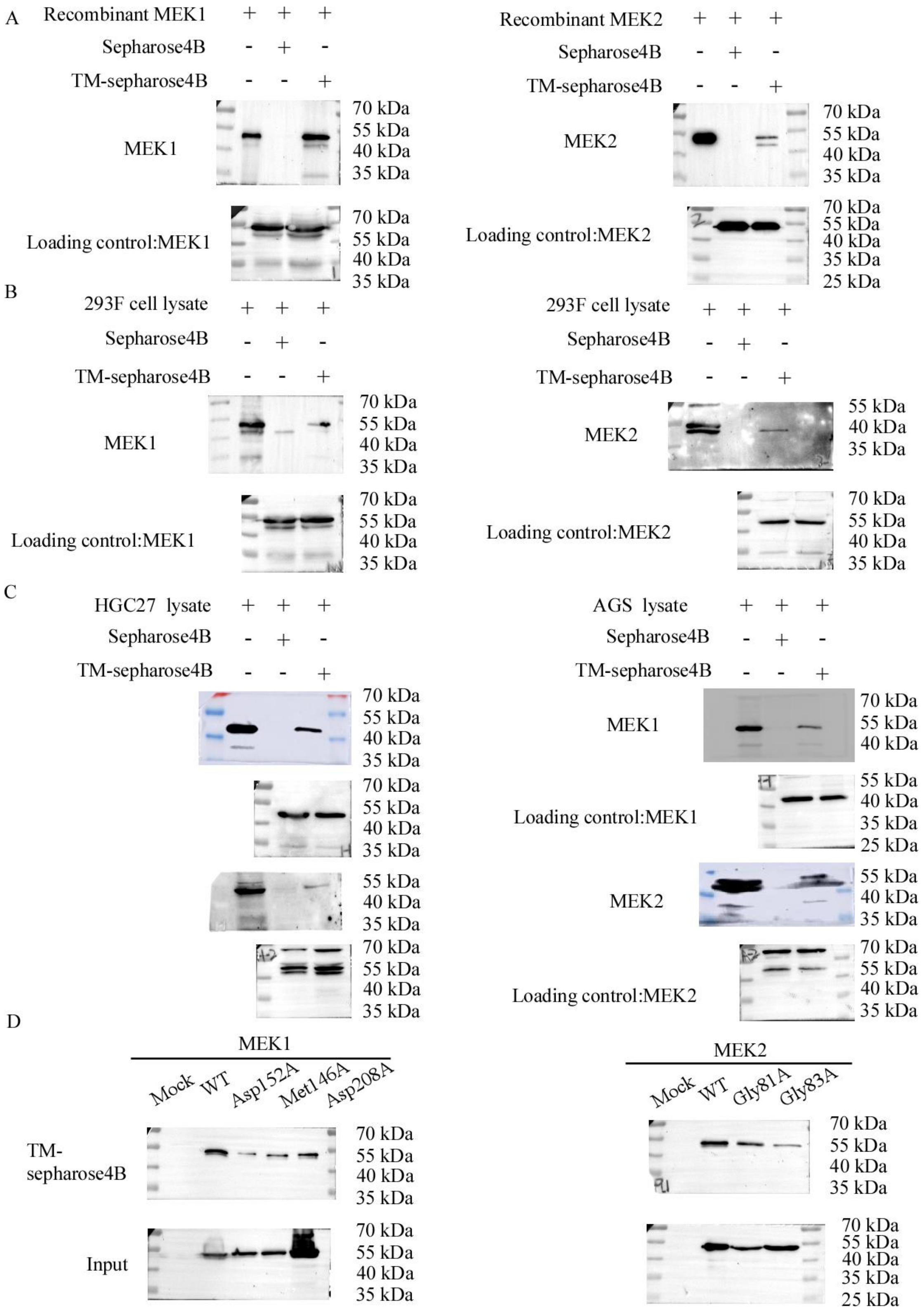

Figure S6. Original blots of Figure 2. The binding ability of tegaserod maleate to recombinant MEK1 and MEK2 protein (A), overexpressed MEK1 and MEK2 protein in 293F cells (B) and endogenic MEK1 and MEK2 protein in vitro (C), obtained via pull-down assay. (D) The binding ability of tegaserod maleate to mutant MEK1 and MEK2.

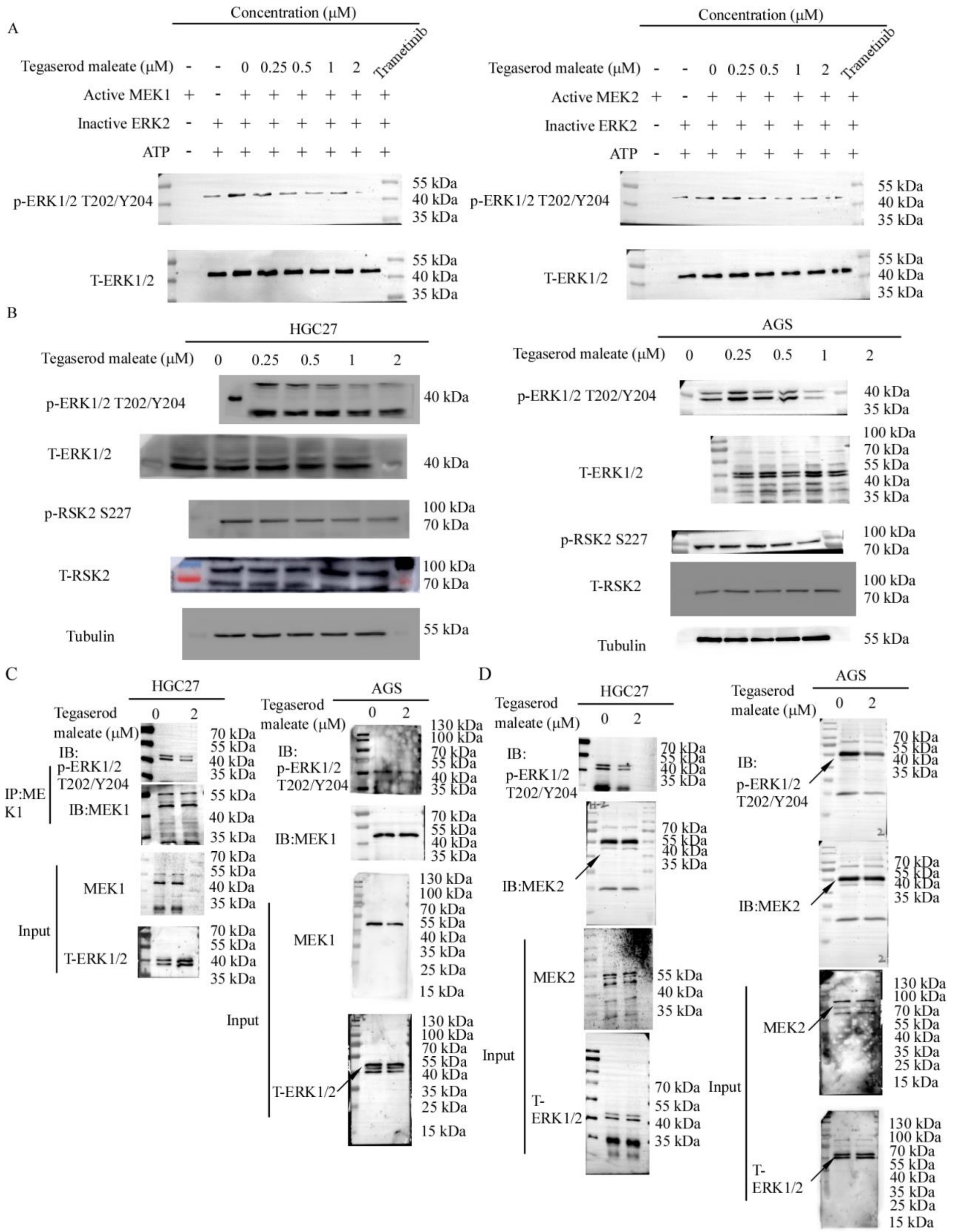

Figure S7. Original blots of Figure 3. (A) MEK1 and MEK2 kinase activity was assessed by in vitro kinase assay using active MEK1, MEK2 and inactive ERK2 proteins. The effect of tegaserod maleate was determined using Western blotting. (B) The levels of p-ERK1/2, ERK1/2, p-RSK2 and T-RSK2 in HGC27 and AGS cells with different concentrations of tegaserod maleate (0, 0.25, 0.5, 1 and 2 μM) treatment for 24 h was determined by Western blotting. (C) The levels of p-ERK1/2 T202/Y204 were affected by MEK1 and MEK2 in HGC27 (C) and AGS (D) when treated with tegaserod maleate. ERK1/2 was immunoprecipitated by MEK1/2 and ERK1/2 was detected using p-ERK1/2 T202/Y204.

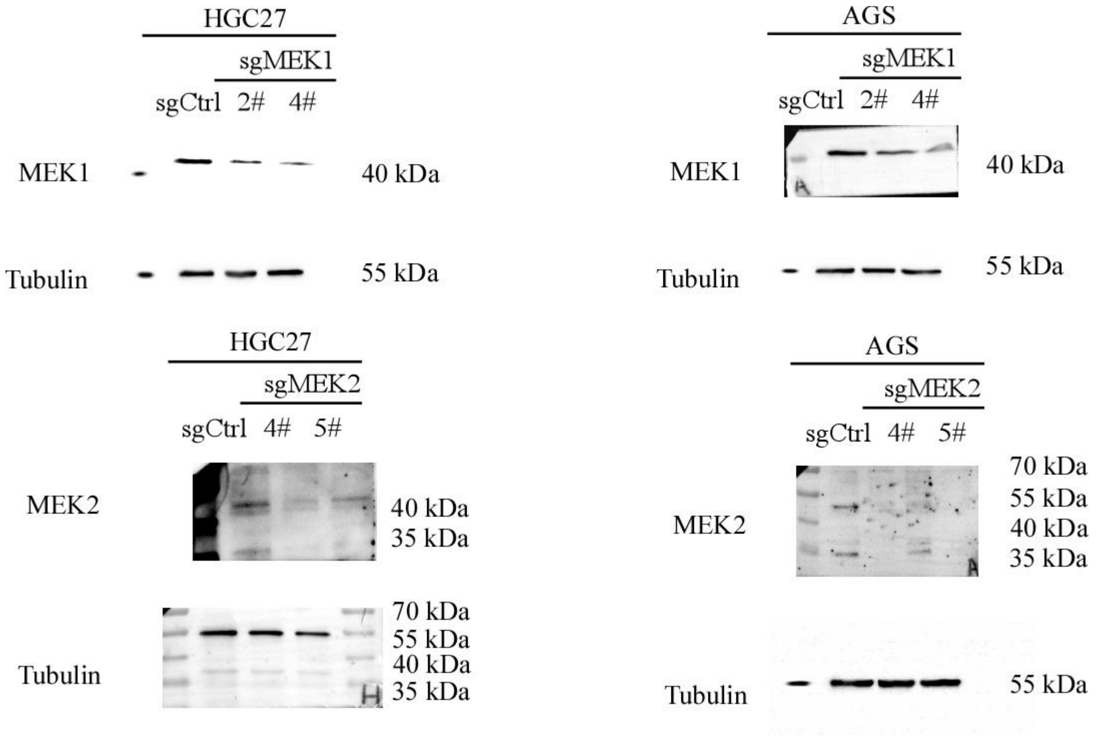

Figure S8. Original blots of Figure 4. The knockout efficiency of MEK1 and MEK2 was verified by Western blotting. Tubulin served as loading control.

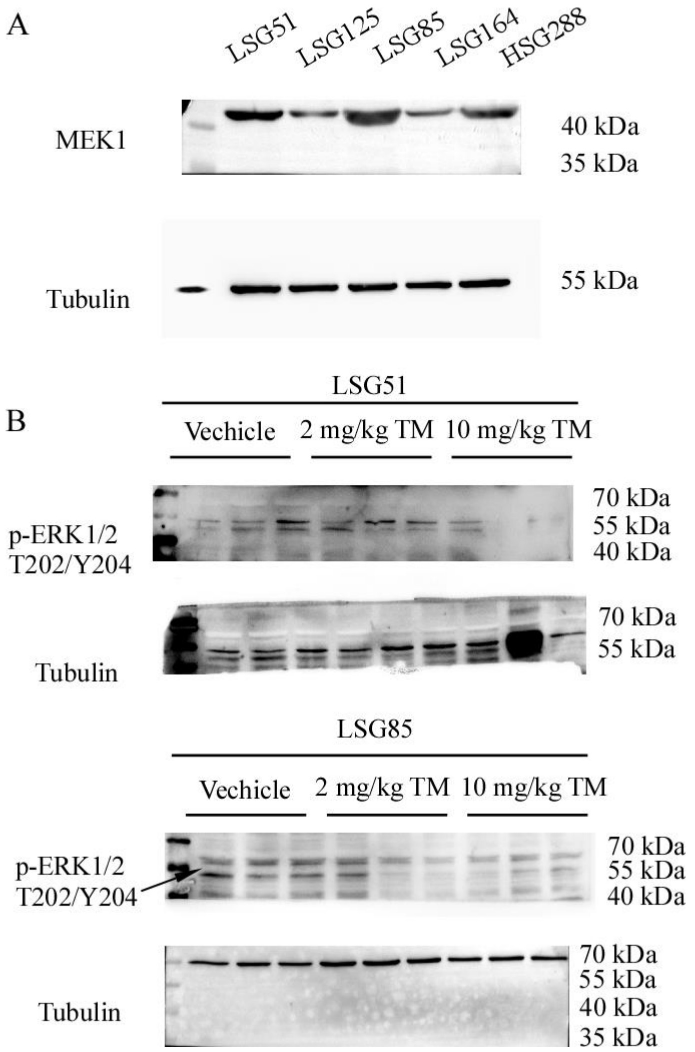

Figure S9. Original blots of Figure 5. (A) We screened tumor cases with high MEK1 expression by Western blotting. (B) We investigated the levels of p-ERK1/2 T202/Y204 in tumor tissues by Western blotting. Tubulin served as loading control.

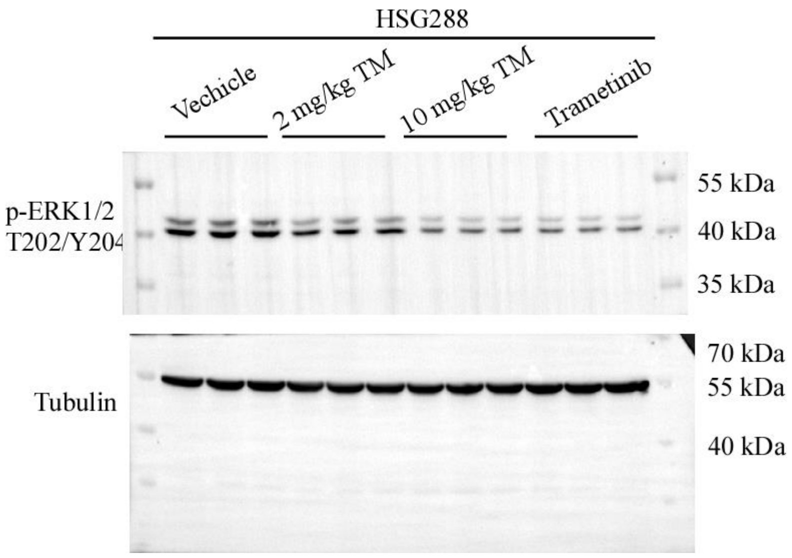

Figure S10. Original blots of Figure 6. We investigated the levels of p-ERK1/2 T202/Y204 in tumor tissues by Western blotting. Tubulin served as loading control.

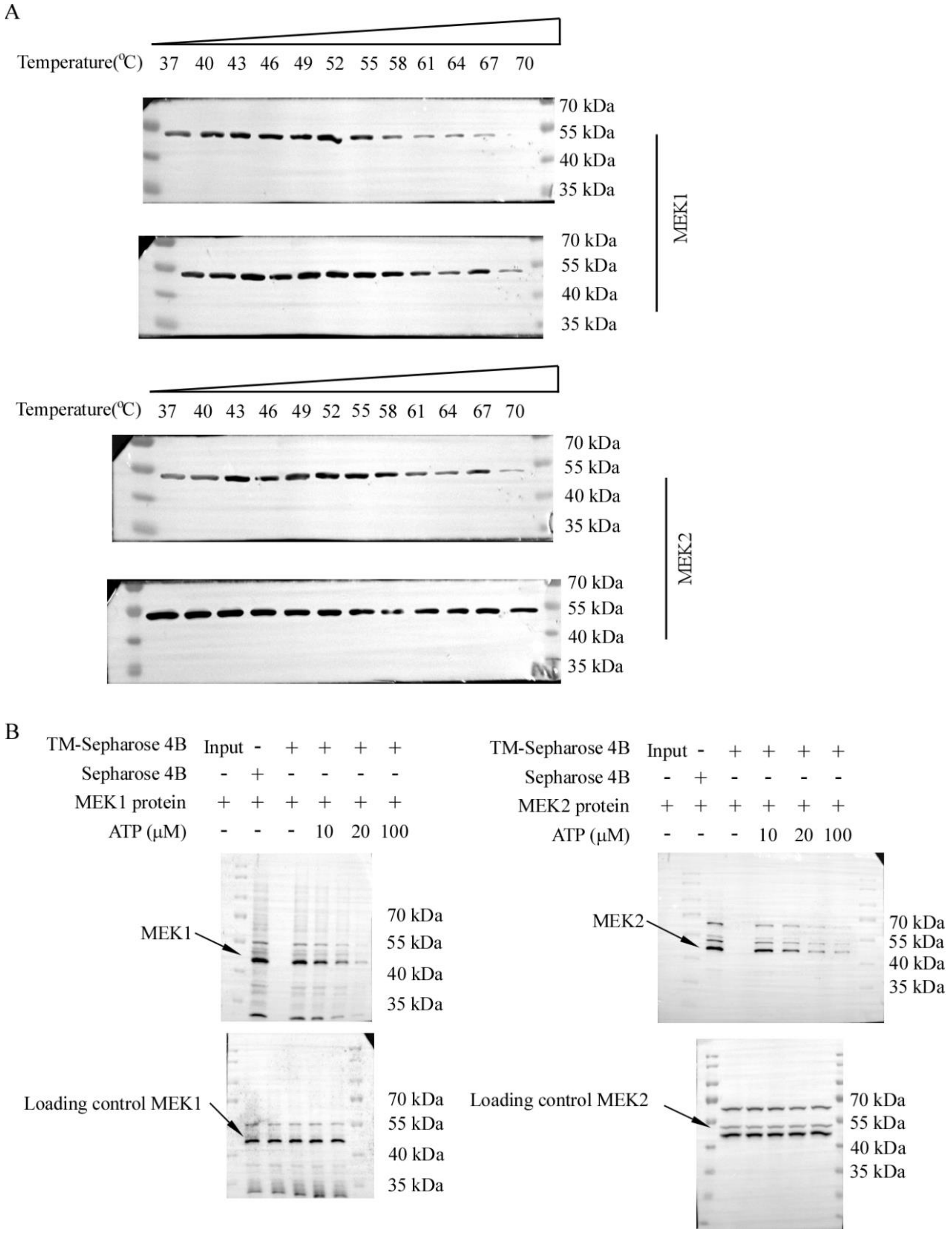

Figure S11. Original blots of Figure S2 (A) The binding of tegaserod maleate to MEK1 and MEK2 proteins was verified by CETSA. (B) The specificity of the binding of tegaserod maleate to active MEK1 and MEK2 in the presence of ATP was evaluated.
